# News and Events of the Week

**Published:** 1910-11-26

**Authors:** 


### News and Events of the Week.

The ordinary general meeting of the Röntgen Society will be held on Thursday, December 1, 1910, at 20 Hanover Square, at 8.15 p.m., when a paper will be read on "Osmotic Growths" by Dr. Deane Butcher.

The winter session of the General Medical Council opened at the offices in Oxford Street on Tuesday, when Sir Donald MacAlister, the President, delivered an address. Sir Isambard Owen, the new representative of the University of Bristol, was introduced by Dr. Norman Moore.

The following are the arrangements at the Central London Throat and Ear Hospital, Gray's Inn Road, London, W.C. : Lectures—Tuesday, November 29, at 3.45 p.m., "Methods of Examination," Dr. Dundas Grant; Friday, December 2, at 3.45 p.m., "Methods of Examination," Dr. Dundas Grant.

Mr. Benjamin Wainewright, M.B., C.M., F.R.C.S., of 104 Park Street, Grosvenor Square, W., consulting ophthalmic surgeon to the London Medical Aid Society, surgeon to the Royal Westminster Ophthalmic Hospital and to the Charing Cross Hospital, who died at Pontresina, Switzerland, on August 28, aged fifty-seven, left estate in the United Kingdom valued at £114,478 gross, and £47,021 net.

According to *Le Journal* it will not be long before we are deprived of the amusing travellers' tales told with regard to the practices which pass for therapeutics among the Chinese practitioners of to-day. Our American cousins have taken the matter in hand, and there is to be an end of the cabalistic incantations, mysterious sachets, powders of horrible composition, and the rest of the marvels of an oriental pharmacopœia. Several doctors of Harvard University have decided to found a large medical school for Chinese students, and Shanghai has been chosen as a place suitable for building such an institution. It is said that the project has been received with great approbation by many influential Chinese. It only remains to us to bestow our blessing on this crusade of science and hygiene against the prejudices of an ancient civilisation.

On November 16 the Right Hon. Lord Winterstoke opened the chemical and physiological wing at the Bristol University. The scene in the great hall was impressive, and the robes worn by the Lord Mayor, Aldermen, and others added colour and beauty. Among those present were the Lord Mayor and Lady Mayoress, the Duchess of Beaufort, the Bishop of Bristol, the Vice-Chancellor and the pro-Vice-Chancellor, Professor Lloyd Morgan, members of the University Council and staff, members of the City Council, and many distinguished visitors. There was a short service in the Hall, followed by an address by Lord Winterstoke. A procession was formed, and moved to the main entrance of the new wing. After the Lord Mayor's speech, Lord Winterstoke opened the door with a silver key. About 1,500 guests were entertained in the evening at a conversazione by the Right Hon. Lewis Fry and the Council of the University. Many interesting demonstrations were made, the various departments making their special exhibits, and a most interesting and enjoyable evening was spent.

Dr. Squire Sprigge's book on "Medical Education," reviewed in last week's issue of The Hospital, is published by Baillière, Tindall, and Cox, 8 Henrietta Street, Covent Garden, London, and not by Bale, Son, and Danielsson, as specified in the review.

Mrs. James Wylie, widow of the owner of the Château d'Eilenroc at Cap d'Antibes, has informed the Mayor of Antibes, says the *Times* correspondent, through the medium of the British Vice-Consul at Cannes, that she places at his disposition the sum of £20,000 for the construction of a new hospital at Antibes.

The following are the arrangements for next week at the West London Post-graduate College : November 28 and December 1, demonstrations by Surgical Registrar, at 10 a.m. ; December 2, demonstration by Medical Registrar, at 10 a.m. ; November 29, demonstration of minor operations, at 11.30 a.m. ; November 28, pathological demonstration, at 12 noon; November 30, lecture, Dr. Grainger Stewart, practical medicine, at 12.15 p.m. Lectures at 5 p.m. : November 28, Mr. Dunn on "Purulent Conjunctivitis in the Infant and Adult"; November 29, Medical Registrar, "Gastric Ulcer"; November 30, Dr. Morton on "X-Ray Examination of the Urinary System"; December 1, Mr. Baldwin on "Practical Surgery"; December 2, Mr. Richard Lloyd on "Anæsthetics."

The following are the arrangements at the Medical Graduates' College and Polyclinic, 22 Chenies Street, W.C. : Monday, November 28, at 4 p.m., Dr. J. L. Bunch, Clinique (skin); at 5.15 p.m., Dr. J. H. Stowers, "Drug Eruptions : their Nature and Varieties." Tuesday, November 29, at 4 p.m., Dr. James Taylor, Clinique (medical); at 5.15 p.m., Dr. T. D. Lister, "Hæmoptysis." Wednesday, November 30, at 4 p.m., Mr. James Berry, Clinique (surgical); at 5.15 p.m., Dr. F. W. Price, "The Early Diagnosis of Pulmonary Tuberculosis." Thursday, December 1, at 4 p.m., Mr. L. H. McGavin, Clinique (surgical); at 5.15 p.m., Dr. James Galloway, "On Arterio-Sclerotic Degenerations of the Skin." Friday, December 2, at 4 p.m., Dr. Dan Mackenzie, Clinique (ear, nose, and throat).

The Lord Mayor of London will open a Conference of Health-promoting Institutions, to be held at the Guildhall on December 8 and 9, which is being organised by the National League for Physical Education and Improvement. The subjects and speakers at the three sessions are as follows : (1) How to work a "School for Mothers" (Lady Meyer, Vice-President of the St. Pancras "School"); (2) Infant Welfare Schemes Abroad (Miss H. M. Blagg); (3) Day Nurseries (Muriel Viscountess Helmsley, Chairman of the National Association of Day Nurseries); (4) What may be accomplished by Children's Care Committees (Miss M. Frere, member of the Education Committee of the L.C.C.); (5) Health Societies : their Aims and Opportunities (Mr. Douglas Eyre); (6) The Co-ordination of Health-promoting Agencies (Mr. F. E. Fremantle, County Medical Officer of Health for Herts). The chair will be taken at the various sessions by Alderman Broadbent, of Huddersfield, the Duchess of Marlborough, Mr. Whitaker Thompson (Chairman of the L.C.C.), and Sir Shirley Murphy (Medical Officer of Health to the L.C.C.). Tickets of admission may be obtained gratis on application to the Secretary of the League, at 4 Tavistock Square, W.C.

